# Report on the International Colloquium on Cardio-Oncology (Rome, 12–14 March 2014)

**DOI:** 10.3332/ecancer.2014.433

**Published:** 2014-05-29

**Authors:** Michael Ewer, Luca Gianni, Fabrizio Pane, Maria Teresa Sandri, Rudolf K Steiner, Leszek Wojnowski, Edward T Yeh, Joseph R Carver, Steven E Lipshultz, Giorgio Minotti, Gregory T Armstrong, Daniela Cardinale, Steven D Colan, Sarah C Darby, Thomas L Force, Leontien CM Kremer, Daniel J Lenihan, Stephen E Sallan, Douglas B Sawyer, Thomas M Suter, Sandra M Swain, Flora E van Leeuwen

**Affiliations:** 1 The University of Texas MD Anderson Cancer Center, Houston, TX 77030, USA; 2 San Raffaele Hospital, Milan 20132, Italy; 3 Federico II University School of Medicine, Naples 80131, Italy; 4 European Institute of Oncology, Milan 20141, Italy; 5 University of Zurich, Zurich 8006, Switzerland; 6 University of Mainz, Mainz 55099, Germany; 7 University of Pennsylvania and Abramson Cancer Center, 1600 Penn Tower, 3400 Spruce St, Philadelphia, PA 19104, USA; 8 Wayne State University and Children’s Hospital of Michigan, 3901 Beaubien Blvd, Detroit, MI 48201, USA; 9 University Campus Bio-Medico, Via Alvaro del Portillo 21, Rome 00128, Italy; 10 Working Group of the International Colloquium on Cardio-Oncology (St Jude Children’s Research Hospital, Memphis, TN 38105, USA); 11 Working Group of the International Colloquium on Cardio-Oncology (European Institute of Oncology, Milan 20141, Italy); 12 Working Group of the International Colloquium on Cardio-Oncology (Harvard Medical School, Boston, MA 02115, USA); 13 Working Group of the International Colloquium on Cardio-Oncology (University of Oxford, Oxford OX3 7LF, UK); 14 Working Group of the International Colloquium on Cardio-Oncology (Vanderbilt University School of Medicine, Nashville 37232, TN, USA); 15 Working Group of the International Colloquium on Cardio-Oncology (Emma Children’s Hospital-Academic Medical Center, Amsterdam 1100 DD, The Netherlands); 16 Working Group of the International Colloquium on Cardio-Oncology (Dana-Farber Cancer Institute, Boston, MA 02215, USA); 17 Working Group of the International Colloquium on Cardio-Oncology (Bern University Hospital, Bern 3012, Switzerland); 18 Working Group of the International Colloquium on Cardio-Oncology (Washington Cancer Institute, Washington, DC 20010, USA); 19 Working Group of the International Colloquium on Cardio-Oncology (Netherlands Cancer Institute, Amsterdam 1066 CX, The Netherlands)

**Keywords:** cardio-oncology, antitumor therapies, cardiovascular sequelae, patients, survivors

## Abstract

Cardio-oncology is a relatively new discipline that focuses on the cardiovascular sequelae of anti-tumour drugs. As any other young adolescent discipline, cardio-oncology struggles to define its scientific boundaries and to identify best standards of care for cancer patients or survivors at risk of cardiovascular events. The International Colloquium on Cardio-Oncology was held in Rome, Italy, 12–14 March 2014, with the aim of illuminating controversial issues and unmet needs in modern cardio-oncology. This colloquium embraced contributions from different kind of disciplines (oncology and cardiology but also paediatrics, geriatrics, genetics, and translational research); in fact, cardio-oncology goes way beyond the merging of cardiology with oncology. Moreover, the colloquium programme did not review cardiovascular toxicity from one drug or the other, rather it looked at patients as we see them in their fight against cancer and eventually returning to everyday life. This represents the melting pot in which anti-cancer therapies, genetic backgrounds, and risk factors conspire in producing cardiovascular sequelae, and this calls for screening programmes and well-designed platforms of collaboration between one key professional figure and another.

The International Colloquium on Cardio-Oncology was promoted by the Menarini International Foundation and co-chaired by Giorgio Minotti (Rome), Joseph R Carver (Philadelphia, Pennsylvania, United States), and Steven E Lipshultz (Detroit, Michigan, United States). The programme was split into five sessions of broad investigational and clinical relevance (what is cardiotoxicity?, cardiotoxicity in children, adolescents, and young adults, cardiotoxicity in adults, cardiotoxicity in special populations, and the future of cardio-oncology). Here, the colloquium chairs and all the session chairs briefly summarised what was said at the colloquium. Topics and controversies were reported on behalf of all members of the working group of the International Colloquium on Cardio-Oncology.

## What is cardiotoxicity?

Thomas Force (Vanderbilt University, Nashville, TN) opened this session and presented basic studies that showed how anti-cancer drugs could affect the heart. He showed that anthracyclines induce cardiotoxicity by multiple mechanisms (e.g., by targeting topoisomerase 2β), whereas trastuzumab binds to erbB2 receptor to blunt pro-survival signals that help cardiomyocytes to withstand chemical or hemodynamic challenges. On the other hand, sunitinib prevents cardiomyocytes from responding to stress by blocking the PDGF receptor. These are reasonably well-defined mechanisms for cardiotoxicity that rest with good correlations between animal models and clinical observation. However, Force showed how pre-clinical models may not always be adequate to truly predict cardiotoxicity with the small molecule kinase inhibitors or to define mechanisms of injury from these drugs. The disconnection between pre-clinical models and patient outcomes could be due to the lack of ‘confounding’ conditions in the animal models. Many cancer patients undergoing chemotherapy have co-morbid conditions, such as coronary artery disease, hypertension, and diabetes. These confounders were not present in the animal models used to study cardiotoxicity. To fill this gap, Force and his colleagues created myocardial infarction in their mouse model before treating them with sorafenib. They found that mice with myocardial damage have marked reduction in survival when sorafenib was added. These results were conceptually expected, highlighted the importance of co-morbidities in the development of cardiotoxicity, and showed the need for translational models that incorporate co-morbidities. To emphasise how complex this scenario can be, Force also showed that the off-target effect of tyrosine kinase inhibitors is not limited to inhibiting kinases in adult cardiomyocytes; he showed that the anti-leukaemic drug, imatinib, blocks proliferation of c-Kit positive side-population cardiac stem cells via off-target binding to breast cancer resistance protein.

Force concluded that better strategies are needed to overcome the limitations or pitfalls of the current approaches to detecting cardiotoxicity. In addition to the rodent model, zebra fish can serve as an important platform to detect cardiotoxicity from investigational or approved drugs. In clinical settings, new biomarkers should be developed and validated vis-à-vis the information offered by troponin I (cfr. contribution by Daniela Cardinale later in this report). In particular, the use of metabolomics seems to open up new avenues to filling the gap between pre-clinical and clinical models.

Steven Colan (Harvard University, Boston, MA) expanded Force’s concepts to rethink a definition of cardiotoxicity in patients. He introduced a concept called ‘actionable’ cardiotoxicity, which implies some management change in response to clinical findings or patient’s symptoms may be warranted. Actionable cardiotoxicity should be distinguished from toxicity definitions that often include alterations of no proven consequence. He stated that adverse events detected during the course of anthracycline chemotherapy may not always have long-term consequences. For example, early investigations into changes in myocardial biopsy demonstrated cardiotoxicity before 180 mg/m^2^, with a dose-related increase in these histologic abnormalities, but these biopsy findings correlated poorly with clinical manifestations and outcomes. In contrast to the biopsy findings, the cumulative dose-related cardiomyopathy associated with doxorubicin demonstrates remarkable variability amongst individuals. To date, virtually all of the progress in toxicity reduction has been achieved by global reduction of cumulative drug dosage and co-administration of cardioprotectant agents, such as dexrazoxane. Colan focused his discussion of ‘actionable’ cardiotoxicity on the best studied class of chemotherapy agents, the anthracyclines. Anthracycline cardiotoxicity can be either early onset or late onset (one year following therapy). Left ventricular strain has increasingly been touted as a superior indication of cardiotoxicity as compared with the old standard, left ventricular ejection fraction. However, the published strain studies did not take into consideration the loading conditions and did not provide long-term outcome correlation. Cardiac magnetic resonance imaging (MRI) has confirmed prior echocardiographic observations of high incidence of elevated wall stress with diminished mass/volume as a predictor of congestive heart failure (CHF), arrhythmias, and death. That being said, its predictive value for late outcome is unknown. Furthermore, the predictive value of higher mean extracellular volume (indicator of diffuse fibrosis) as detected by MRI is also unknown.

There are two major ways to prevent anthracycline-induced cardiotoxicity. The first is primary prevention that does not require monitoring during therapy. The strategies for primary prevention include limitation of dose, continuous infusion, liposomal preparation, or co-administration of the only US Food and Drug Administration approved cardioprotectant, dexrazoxane. The second approach is to monitor the patients with either imaging tools or biomarkers during therapy and intervene only when toxicity signals appear. If anthracyclines were either dose-reduced or stopped because of clinical or subclinical evidence of cardiotoxicity, the potential downside is inadequate tumour eradication. Colan also argued that cancer therapy is associated with a variety of haemodynamic changes, such as hyper- or hypotension, hyper- or hypovolemia, and changes in the sympathetic or parasympathetic tones. These factors can fluctuate widely and limit the utility of imaging-based strategy for detection of cardiotoxicity in children. He, therefore, concluded that the utility of toxicity ‘definition’ is dependent on the purpose of the classification. Test-driven dose reduction requires a high predictive accuracy for acute or chronic toxicity that may limit survival. High specificity for acute toxicity is of most importance for revision of treatment programmes. High sensitivity for late toxicity is of most importance for modification of recommendations for late monitoring.

During the discussion period, questions about the relevance of pre-clinical testing on real clinical outcome were raised. For example, imatinib was initially shown in animal models and patient studies to be cardiotoxic. However, perhaps due to reduction of dose or better treatment of co-morbidities, imatinib is not considered as a cardiotoxic drug in its current usage. Again this showed the gap between pre-clinical and clinical detection of cardiotoxicity. Following on the concepts of actionable cardiotoxicity and cardioprotection, the group also began the discussion of the lack of use of dexrazoxane. There was a consensus that dexrazoxane should be resuscitated to a wider clinical usage; this was a leitmotif that surfaced in many other discussions at the meeting.

## Cardiotoxicity in children, adolescents, and young adults

Steven Lipshultz (Wayne State University, Detroit, MI) opened this session by stating that advances in cancer treatment have greatly improved childhood cancer survival rates, which are still largely based on anthracycline-based chemotherapies; however, this occurs at the cost of higher rates of heart failure, coronary artery disease, and cerebrovascular events in childhood cancer survivors as compared with controls.

Lipshultz emphasised that anthracycline-induced cardiotoxicity can cause persistent and progressive cardiac damage. Accumulations of mitochondrial DNA mutations and consequent defects of the respiratory chain were considered important determinants of the inexorable progression of anthracycline cardiotoxicity. Lipshultz also cautioned that anthracycline cardiotoxicity should be viewed as a dynamic process that progresses through distinct clinical phenotypes. Cardiotoxicity may transition from an early subclinical dilated cardiomyopathy to a delayed potentially restrictive cardiomyopathy. The restrictive phase manifests as a relative decrease in left ventricle dimension with a geometrically consequent rise in wall thickness, leading to a normal thickness–dimension ratio at latest follow up. There is also a progressive fall in left ventricle mass and cavity size that becomes inadequate for body size (Grinch Syndrome, ‘heart too small for body size’).

Lipshultz highlighted the importance of primary and secondary cardiotoxicity prevention strategies that did not compromise anti-tumour activity. Having described inconsistent cardioprotection from substituting slow infusions for bolus infusion, Lipshultz showed dexrazoxane co-administration as the most doable primary means to prevent anthracycline cardiotoxicity in paediatric cancer patients. He showed that dexrazoxane delayed or mitigated clinical signs and echocardiographic indices of cardiac remodeling, blunted early elevations of circulating markers (troponin and B-type natriuretic peptide), allowed for anthracycline dose escalations. Lipshultz suggested that primary prevention might also extend to identifying genetic factors that increase individual susceptibility to cardiotoxicity. He described associations between mutations of human haemochromatosis gene, early elevations of troponin and natriuretic peptide during anthracycline therapy, greater left ventricle dilation, and dysfunction at two years follow up. In a similar manner, single-nucleotide polymorphisms in drug biotransformation or transport/elimination genes could put patients at a greater risk of cardiotoxicity.

In the settings of secondary prevention and treatment, Lipshultz showed that angiotensin-converting enzyme inhibitors offered only transient improvements in childhood cancer survivors with a diagnosis of left ventricle dysfunction. This emphasised the importance of primary prevention or earlier (prophylactic) treatment. With that said, Lipshultz described the cost-effectiveness of echocardiographic follow-up recommendations by the Children Oncology Group, which allowed for earlier diagnosis, delayed heart failure, and improved quality of life.

Gregory Armstrong (St Jude Children’s Research Hospital, Memphis, TN) discussed the role of chronic health conditions because many long-term survivors of cancer will develop traditional, modifiable risk factors related to ageing, hereditary predisposition, or unhealthy lifestyle behaviours. In the general population, hypertension, diabetes, obesity, dyslipidemia, and smoking are primary contributors to the development of coronary artery disease and heart failure. Because cancer therapies increase the risk for hypertension, diabetes mellitus, dyslipidemia, and obesity, it is imperative to determine the extent that modifiable cardiovascular risk factors further potentiate cancer therapy-associated cardiac risk.

In describing reports from the Childhood Cancer Survivors Study, Armstrong showed that 10% of 50-year-old cancer survivors carry three chronic health conditions, with ~50% and ≥20% cumulative incidence of, respectively, first and second condition. In contrast, age-matched siblings showed only one or two conditions, with ≤20% cumulative incidence of the first condition. Armstrong described that risk for morbidity and mortality continues across the life span for cancer survivors and may help to explain some impressive figures. For example, one in five healthy survivors age 45 had a severe, life-threatening event or death within ten years, as opposed to one in 14 age-matched siblings. Moreover, among some survivors, the observed risk for life-threatening cardiovascular events may be greater than one would expect upon assuming additive interactions between chronic conditions and chemotherapy-related cardiac damage.

Armstrong suggested that clinical implications of these studies are of importance as primary or secondary prevention strategies may substantially reduce the incidence of cardiovascular sequelae in at risk cancer survivors. Among primary prevention measures Armstrong re-emphasised those addressed by Lipshultz. Among secondary prevention measures, Armstrong showed improved participation to special programmes of counselling, follow up, and adherence to guidelines for a healthy lifestyle (e.g., advanced practice nurse, American Institute for Cancer Research Guidelines for a healthy lifestyle).

In his closing remarks, Armstrong stated that: 1) childhood cancer survivors have a significant, life-long risk for cardiotoxicity that is remarkably potentiated by increased incidence of modifiable cardiovascular risk factors, 2) treatment of clinical cardiomyopathy may be of limited success, which is in agreement with Lipshultz’s opinion, 3) prevention should be given priority, and 4) numerous preventive strategies exist but require additional investigation, in particular, randomised trials with extended longitudinal follow up.

Flora van Leeuwen (Amsterdam, The Netherlands) concluded this session by discussing cardiotoxicity from radiotherapy and chemotherapy in adolescent and young adult Hodgkin’s lymphoma survivors. Although cardiovascular events occasionally occur during adolescence or young adulthood, most occur ≥2 decades after the original exposure. Van Leeuwen described studies that identified 1209 cardiovascular diseases in 747 patients after a median follow up of 20.4 years. After mediastinal radiotherapy, the 35-year cumulative incidence of cardiovascular diseases was ~45% compared with ~20% in patients not treated with mediastinal radiotherapy. After anthracyclines, the 30-year cumulative incidence of heart failure was ~16% compared with ~10% in patients not treated with anthracyclines. By saying that radiation may cause damage to cardiac vasculature, valves, and myocytes, van Leeuwen showed that the most frequently diagnosed first cardiac event after radiotherapy was ischaemic heart disease (myocardial infarction), followed by valvular disease, or a second ischaemic disease (angina pectoris). Cardiomyopathy and/or CHF was mostly diagnosed as end-stage cardiovascular disease. Of note, several patients with treatment-related cardiovascular disease developed multiple cardiovascular diseases.

An important task of van Leeuwen’s lecture was to identify patient-related risk factors and treatment-related risk factors. Among the former, van Leeuwen indicated age at diagnosis/treatment, follow-up time, general risk factors for cardiovascular disease, and genetic factors; among the latter, she indicated chemotherapy agents (type and dosages), radiotherapy dose and volume, interactions of radiotherapy with chemotherapy, multiple interactions between radiotherapy, chemotherapy, age, genetics, and general risk factors. Diagnostic splenectomy, obesity, hypertension, hypercholesterolaemia, were identified as treatment- and patient-related risk factors that specifically predisposed to valvular disease. Van Leeuwen also showed that mediastinal irradiation increased the risk of all cardiovascular diseases, while anthracyclines increased the risk of valvular disease and CHF. Possible additive or more than additive interactions between radiation and anthracyclines were discussed.

Screening of Hodgkin’s disease survivors or new patients is highly recommended. Van Leeuwen described the nationwide BETER project that was designed to recall and screen survivors at high risk. For new patients, she suggested improved risk-adapted treatment and prediction models at start of treatment that helped balancing chances of cure versus late effects. Lowering volumes and doses of radiotherapy, or lowering anthracycline doses, might also be considered; and however, these latter measures should be carefully weighed against the risk of depriving patients of life-saving doses of radiation and anthracyclines. Balancing concerns about cardiotoxicity and the need for life-saving full dose therapies became another leitmotif of the colloquium. In particular, van Leeuwen considered that modern radiotherapy techniques enable more accurate sparing of the heart; and however, further development of these techniques is much needed because increased use of systemic treatment and improved survival rates magnify the risk for delayed cardiac events.

## Cardiotoxicity in adults

The session on cardiotoxicity in adults hosted two extremely interesting and closely related presentations. Thomas Suter (Bern, Switzerland) opened the session with a discussion that focused on the differences between how we assess cardiotoxicity in the clinical setting and how that differs in some ways from how we adjudicate a cardiac event in a clinical trial. Once again, Suter pointed out that in the clinical setting our goal is to protect the patient, and doing so requires a balance between the risks of undertreating the malignancy on the one hand, and inducing a cardiac event that could impact on either survival or quality of life on the other. This goal is somewhat different from those used in clinical trials, where the goal is to quantitate the extent of cardiac events for the agent under consideration.

Suter reviewed a novel way to approach cardiac events for any given patient by looking at myocardial function over time. An improving left-ventricular function suggests a more favorable long-term outlook and perhaps a lesser need for high levels of scrutiny in ensuing years. A patient with declining function over time after treatment with a potentially cardiotoxic agent might be re-evaluated more frequently, and may be a candidate for more intense and earlier intervention. The need for consistent criteria to identify both patients with adverse events, as identified in clinical trials or in everyday clinical practice, is crucial. In trials, consistent criteria would allow for meaningful comparisons of different agents, combinations of agents, or administration schedules; while in the clinical setting the risk of under and over inclusion of patients could be minimised.

Daniel J Lenihan (Vanderbilt University, Nashville, TN) stressed the need for cardiac risk stratification. The difficulty in quantifying cardiac dysfunction in asymptomatic patients was brought to the forefront, and the fact that older patients were more likely to have underlying cardiovascular disease was stressed. Such patients, therefore, were more likely to experience further declines in either reserves or function status with exposure to cardiotoxic agents, and were candidates for more aggressive intervention. Maintaining residual function requires optimal management of co-existing conditions, and prudent strategies should have a major impact over the course of active treatment. Lenihan suggested that sophisticated cardiac testing, including biomarkers, cannot replace sound clinical judgment. Active collaboration and discussion among providers from different disciplines, is the future template for the best practice.

Considerable discussion ensued regarding differences in the mechanisms of toxicity and methods for evaluating and treating. Should a patient who has experienced a functional decline presumably related to the use of an anthracycline be managed in the same way as one who experienced a similar decline following the use of, e.g., trastuzumab? While we recognise that functional tests, such as cardiac ultrasound or multigated blood-pool scans (MUGA), cannot (or cannot yet) provide data as to mechanism of dysfunction, many cardiologists treat all such declines equally even though the spontaneous reversibility of impaired function is reportedly higher with non-anthracyclines. Suter, Lenihan, and the working group of the colloquium recognised that data furthering an individual approach, based not only on the degree of dysfunction but also on the characteristics of the agent used, are not yet available. There was general agreement, however, that communication between the various parties treating cancer patients is important, that more uniform criteria for defining cardiotoxicity was essential both for adjudicating events in clinical trials and for managing patients, and that data should be accumulated so that more specific guidance based on underlying cardiac status, agent used, and overall prognosis can be integrated. Only then can data-driven practice guidelines be incorporated into clinical pathways. Cardio-oncology, or onco-cardiology, has made strives, but much work remains to be done.

## Cardiotoxicity in special populations

Joseph Carver (University of Pennsylvania, Philadelphia, PA) opened this session dedicated to the special populations of cancer patients presenting with an added risk of cardiotoxicity. He focused on the elderly. The population is ageing and with it, an ever-increasing amount of cancer in the elderly.

Besides its prevalence, cancer in the elderly presents special challenges because of pre-existing comorbidities, age-related changes in drug pharmacokinetics, altered physical and cognitive function, potential lack of social support, and poorer response rates. Carver stated that these variables require standardised assessment and the risks associated with these non-cardiac factors often outweigh the risk of cardiotoxicity. The elderly are often undertreated because of fear of toxicity leading to treatment that does not have curative intent. Moreover, there is a tendency to ignore that in the elderly, cardioprotective strategies may have the same benefit as in younger patients. In addition, the lack of enrolment of older patients with existing comorbidities, such as cardiac disease, in clinical trials has limited the accumulation of evidence-based clinical knowledge that is sorely needed.

According to Carver, the constant increase in cancer incidence and the projected explosive growth of the older population over the next 25 years are compelling forces driving the scientific community to answer critical questions of cancer care delivery for the future. These include, but are not limited to, the following: 1) can we accurately predict in the elderly as we are doing in the young adult, who will develop chemotherapy-related cardiotoxicity?, 2) can patients with underlying heart disease receive anthracycline-based chemotherapy or other potentially cardiac toxic but highly curative chemotherapy?, 3) how should we monitor and manage patients with cardiotoxicity? Carver also asked whether there is an assessment tool that can be specifically applied and validated to this population for cardiotoxicity. It is, in fact, recommended that a cardiac risk assessment tool be developed and validated, that clinical trials include an elderly subset, and work begins to develop an international data-base to track and help understand the cardiac risk(s) of cancer treatment in this population.

Douglas Sawyer (Vanderbilt University, Nashville, TN) followed on from Carver’s remarks by stating that increasing numbers of patients with cardiovascular disease, such as myocardial infarction and heart failure, are surviving to develop malignancies, and these patients pose a special challenge to their cardiology and oncology providers. Pre-existing cardiovascular disease in a patient newly diagnosed with cancer is likely to change the treatment options offered by some medical and surgical oncologists. A careful cardiac assessment should be done at that time and thoughtful discussion between cardiology and the oncology provider is ideal to develop a collaborative plan that considers both the cardiovascular disease and malignancy history, prognosis, and on-going therapies.

Sawyer cautioned that many commonly used medications for cardiovascular disease may alter the metabolism and transport of cancer therapies, including anthracyclines, and may therefore alter both their anti-tumour and cardiovascular effects. Other cancer treatment-related challenges for the health care team to consider are managing fluid status, neurohormonal blockade, and thromboembolic risk during cycles of cancer therapy that may promote fluid retention, change in haemodynamics, and increase thrombosis or bleeding risk. Some familiarity with the medications used in oncology may improve the likelihood that a patient with cardiovascular disease can be managed effectively during cancer treatment without an exacerbation. Sawyer also considered that when cancer occurs in patients with heart disease, the first question is to assess any imposed limitations in the treatment strategy. The role of the cardiologist is to assess, treat, and stabilise to deliver an optimal patient for cancer treatment and to collaborate with the oncologist in decision making as well as monitoring and managing any cardiac issues during therapy and after its completion. A logical dictum is that ‘the most threatening disease should be treated first’. At times, however, staged approaches to treatment may be necessary or logical to stabilise one disease to enable optimal treatment of the other.

Sawyer’s recommendations on moving forward include: 1) capitalise on the community at the colloquium to develop a learning system, whereby our anecdotes of heart disease patients being treated for cancer might become ‘data’, 2) consider the cardio-oncology movement one of integration, rather than further differentiation, 3) develop institutional strategies to enable formation of local multi-disciplinary teams to improve the outcome of patients with multi-system, or multiple, disease(s).

Most of the concepts outlined by Sawyer were echoed by Carver in his second contribution to this session. Here, he focused on the risk of cardiotoxicity in patients with prior chemotherapy. As a matter of fact, most adult cardiologists know relatively little or nothing about the cardiovascular effects of cancer treatment and assume a pessimistic ‘cancer kills’ attitude in approaching patients during cancer treatment.

Adults, like paediatric patients with prior anthracycline exposure, have an increased lifetime risk of cardiotoxicity and cardiac-related death that increases with cumulative exposure and duration of survival. From a heart failure standpoint they are all American College of Cardiology/ American Heart Association stage B and should be evaluated and treated according to the guidelines to prevent progression to stage C and D. For some large percentage of asymptomatic survivors exposed to anthracyclines, global left ventricle function remains normal but non-ejection fraction abnormalities by advanced imaging can be demonstrated before changes in ejection fraction or clinical symptoms. At present, however, there is no agreement about what to measure, frequency of measurement, treatment response or predictive accuracy for future events. As also mentioned by Colan, novel approaches like strain and others need to be validated more in depth. Finally, no consistent scientifically validated intervention can, or has been shown to, prevent, slow or reverse pre-clinical toxicity.

Although the focus is on late anthracycline cardiotoxicity, there is a significant risk of treated-coincident cardiotoxicity with a long list of targeted agents and the late effects of these agents are to date undefined and unrecognised. As cancer becomes a chronic disease, with layers of varied chemotherapies, there is no understanding (excluding trastuzumab) of the cardiovascular risk of sequential therapies. Cancer treatment is only one cause of late cardiac disease in long-term survivors, and there needs to be a dedicated focus on recognition and treatment of these other competing conditions in this population. Carver concluded with some provocative questions about future developments for minimising the risk of cardiotoxicity in patients with prior chemotherapies. Regardless of possible advances in genetic profiling or pharmaceutical re-engineering of drugs, priority was given to better education at every level about cardio-oncology and to cooperation and communication between the two specialties and other clinical or translational disciplines.

Sarah Darby (University of Oxford, Oxford, UK) followed on from van Leeuwen’s lecture and focused on the risk of cardiotoxicity from radiotherapy in adult patients. Radiotherapy for breast cancer often involves some incidental exposure of the heart to ionising radiation. The effect of this exposure on the subsequent risk of ischaemic heart disease has been documented.

Darby presented data from a population-based case-control study of major coronary events (i.e., myocardial infarction, coronary revascularisation, or death from ischaemic heart disease) in 2168 women who underwent radiotherapy for breast cancer between 1958 and 2001, in Sweden and Denmark. Left breast radiotherapy was followed by a higher rate of major coronary events that right-sided treatment. For each woman, the mean radiation doses to the whole heart and to the left anterior descending coronary artery were estimated from her radiotherapy chart. The overall average of the mean doses to the whole heart was 4.9 Gy and Darby showed that the rate of major coronary events increased linearly with the mean dose to the heart by 7.4% per gray, with no apparent threshold. The increase started within the first five years after radiotherapy and continued into the third decade after radiotherapy. The proportional increase in the rate of major coronary events per gray was similar in women with and women without cardiac risk factors at the time of radiotherapy; however, women with pre-existing cardiac risk factors had greater absolute increases in risk from radiotherapy than other women.

Additional randomised data were presented from the Early Breast Cancer Trialists Collaborative Group (EBCTCG) and additional non-randomised observational data from the Collaborative Group on Observational Studies of Breast Cancer Survivors (COBS). With regard to cardiomyopathy, pericardial or valve disease, and cardiac death, Darby showed that the risk was also increased, but the balance between cardiovascular complications and absolute risk of death from cancer favoured treatment for the vast majority of women. Again, this denoted the importance of delivering life-saving doses of an anti-tumour therapy.

Darby concluded by stating that for each cardiovascular risk, there was substantial variation between countries. This may reflect differences in radiotherapy techniques use and calls for further analyses in the future.

## The future of cardio-oncology

This final session was aimed at taking a look into the future of cardio-oncology. Expert speakers were asked to re-address questions that had been explored by other members of the working group and were felt to represent the forefront of modern cardio-oncology: could institutional or multi-institutional databases help to improve current methodologies for recalling and screening cancer survivors? Do we have cost-effective and easy-to-measure markers for identifying at-risk patients before or during chemotherapy so as to tailor cancer treatment to individual risk factors and/or to commence cardiovascular medications with prophylactic or curative intent?

Stephen E Sallan (Dana-Farber Cancer Institute, Boston, MA) opened this session by stating that whatever we do for preventing or screening cardiotoxicity, it should not be at the cost of undertreating patients or depriving them of drugs that cause cardiotoxicity but save lives. This is why Sallan liked ‘onco-cardiology’ better than ‘cardio-oncology’, which was his way of emphasising that treatment efficacy always comes first. This having been said, Sallan moved back to presentations by Armstrong and van Leeuwen on the long-term screening of childhood cancer survivors. He showed the different power of multi-institutional versus single-institution initiatives (Childhood Cancer Survivor Study, ~25,000 patients; St. Jude Lifetime Cohort Study, ~2000 patients; Emma Children’s Hospital, ~1400 patients).

Sallan next focused on the Dana–Farber Acute Lymphoblastic Leukemia (ALL) Consortium Database (200 patients in a randomised clinical trial of cardioprotection, 550 patients on cardioprotection with dexrazoxane, and 115 patients in long-term follow up). He showed that the consortium was empowered enough to reach the following fundamental information: 1) cardiac toxicity from anthracyclines is the major late effect in ALL survivors, 2) dexrazoxane prevented both acute cardiotoxicity (troponin elevations) and echocardiographic abnormalities at three years follow up, and 3) dexrazoxane did not expose survivors to an increased risk of secondary neoplasm. These results confuted unjustified concerns by regulatory agencies and firmly concluded that dexrazoxane is both protective and safe. Based on the Dana–Farber ALL Consortium experience, Sallan also recommended that survivors be serially evaluated by echocardiography to intercept early manifestations of the Grinch syndrome, even in absence of clinical signs of heart failure. This echoed similar conclusions by Lipshultz.

In concluding his presentation, Sallan stated that lessons from childhood cancer survivors databases and (multi) institutional screening programmes urge for educational programmes that make paediatric oncologists aware of cardioprotection and alert adult cardiologists to late consequences of chemotherapy.

Sandra Swain (Washington Cancer Institute, Washington, DC) followed on from Sallan by illustrating data from the SEER (Surveillance, Epidemiology, End Results) nine areas programme of the US National Cancer Institute, covering approximately 10% of the US population. According to this survey, survival rates for cancers have improved significantly since the 1970s, due largely to earlier detection and/or advances in treatment. Medical and scientific communities are therefore witnessing cohorts of adult cancer survivors exposed to both common morbidities and clinical or subclinical sequelae of cancer treatment. So far, however, what was done to monitor childhood cancer survivors was not duplicated and developed to monitor adult cancer survivors.

In reviewing major trials in the settings of adjuvant breast cancer treatment, Swain concluded that anthracyclines introduced a measurably higher risk of cardiotoxicity compared to nonanthracycline regimens. This calls for long-term follow up and outcome assessment of breast cancer survivors. Swain showed how this problem is being addressed by the US National Surgical Adjuvant Breast and Bowel Project (NSABP)-B31 Long-Term Cardiac (LTC) follow-up study. This recruits participants in NSABP-B31 study who had 18-month cardiac evaluation by MUGA and remain disease-free. The primary outcome of NSABP-B31 LTC rests with MUGA evaluation over a five- to ten-year window from baseline. Secondary outcome rests with retrieving cardiac medication history, comorbid conditions, and patient-reported outcomes. Sample size is 900 patients. In discussing this meritorious project, which was launched in September 2010, Swain commented that retrieving data from participants in clinical trials will only help to screen 3% of cancer patients; the other 97% of care provided is locked up in electronic medical records.

Swain stated that a breakthrough experience may be offered by the American Society of Clinical Oncology project called CancerLinQ (Learning Intelligence Network for Quality). This is a technology system which will interact with any health information technology product, registries, or other ‘big data’ systems. Data will be aggregated and analysed with the aim of improving the quality of care for every patient. In concluding with a look into the future, Swain said that aggregating data within CancerLinQ will allow the oncology community to learn in a much more rapid fashion, and therefore improve outcomes of cancer patients.

The second part of this session dealt with an early identification of patients at risk for cardiotoxicity from cancer treatments. If identified early enough, such patients could be subjected to alternative treatments (pending risk: benefit assessment), to preventive measures, to drugs seemingly slowing the progression to heart failure. Most progress has been achieved with genetic markers and with the marker of myocardial injury troponin.

The status of genetic studies of anthracycline-induced cardiotoxicity was summarised by Leontien Kremer (Emma Children’s Hospital-Academic Medical Center, Amsterdam, The Netherlands). Since 2005, the importance of gene variants has been addressed in a dozen case-control studies. Due to limited size and, thereby, power, all but one genome-wide study investigated variants of limited numbers of candidate genes. Taking as a validation criterion the association of one and the same gene in at least two studies, anthracycline-induced cardiotoxicity appears at least in part be driven by anthracycline pharmacokinetics and by oxidative stress. The former is represented by both outward (ABCC1 and ABCC2) and inward transporters (SLC28A3) and by the drug metabolising enzymes carbonyl reductases 1 and 3 (CBR1-3) and UDP-glucuronosyltransferase 1-6 (UGT1A6). The latter comprises variants of the NADPH oxidase, human haemochromatosis gene, and hyaluronidase synthase 3. Although biochemical evidence supports most of the replicated associations, it should be cautioned that it played a major role in the selection of genes for genotyping. More important is the remarkably high number of associations replicated despite small study sizes and methodological heterogeneity, including limited correction for multiple testing and for confounders.

Kremer described two predictive models that comprised some of the replicated gene variants and clinical risk factors and discriminated better between cases and controls than clinical risk factors alone. However, these encouraging effect models turned out to be cohort-specific, due to differences in genetic associations in these cohorts. Kremer stressed that further progress critically depends on the development of larger and methodologically better harmonised cohorts of childhood cancer survivors. Such cohorts are expected to emerge from PanCareSurFup, a European Union-funded project focusing on the most serious complications of cancer therapies in long-term childhood cancer survivors. PanCareSurFup aims to follow some 80,000 survivors of childhood and adolescent cancer, making it the largest study of its kind to date.

Daniela Cardinale (European Institute of Oncology, Milan, Italy) talked about the detection of patients at risk of cardiotoxicity before the development of symptomatic heart failure or of asymptomatic decrease in left ventricular ejection fraction using circulating biomarkers. Troponin is the gold standard biomarker for necrotic myocardial injury from any cause. Its evaluation during high-dose chemotherapy allowed for early identification and preventive treatment of patients at risk for post-chemotherapy cardiac dysfunction. Cardinale showed that an increase in this marker may help to identify patients needing for more close follow up or preventive strategies, such as the administration of angiotensin converting enzyme inhibitors. Indeed, prophylactic treatment with enalapril of patients with early troponin increase after chemotherapy seems to prevent cardiac dysfunction and associated cardiac events not only in high-dose anthracycline-treated patients, but also in patients treated with standard-dose anthracyclines or newer anti-tumour agents.

In sharing discussions with the working group Cardinale made the point that some difficulties remain in the clinical application of this approach, mainly related to limited comparability of the results obtained with different troponin assays, which differ in analytical performance and in the related cut-off values. Another issue still to be resolved is the appropriate time-point of sampling during chemotherapy. Cardinale stated that these problems might hopefully be addressed and resolved by the ICOS-ONE study, which is a multi-centre prospective randomised trial on the prevention of anthracycline-induced cardiotoxicity based on troponin monitoring.

Cardinale next focused on natriuretic peptides, which have drawn considerable attention in the last years. Different studies suggest that the persistent elevation of B-type natriuretic peptide, or of the aminoterminal fragment of its precursor, is associated with cardiotoxicity, but the evidence is somehow limited by the fact that many studies are underpowered, different methods have been used, different peptides studied and the cardiac endpoints are not shared among studies. Having these considerations in mind, Cardinale discussed the possibility of a multi-marker approach, which could be implemented both during the chemotherapy and in the follow up of patients.

## Conclusions and perspectives

The major goal of the International Colloquium on Cardio-Oncology was to avoid generic statements on the risk of drug-related cardiovascular events in cancer patients. Instead, the working group attempted to draw firm conclusions on what is supported by numbers and figures, asked what could be done to obtain more data and to approach the level of evidence in other settings, suggested strategies for improving collaborations between cardiologists, oncologists, and other stakeholders of modern cardio-oncology (from laboratory researchers to epidemiologists and many other professional figures). In exploring new frontiers of cardio-oncology, the working group of this colloquium loyally concluded that much of our current knowledge is based on clinical use of anthracyclines that have been around for some 40 years. It follows that generalisations on cardiotoxicity, from definitions to clinical monitoring and prevention or treatment, should be avoided. And anthracycline cardiotoxicity itself may prove to be more complex and dynamic than shown in textbooks or papers. This is why concerns about limitations in the clinical use of dexrazoxane recurred so often in presentations and discussions. However, the colloquium moved beyond old drugs and offered a forum for engaging discussions on new therapies and tools in approved and investigational settings. In examining one scenario or another, the working group always balanced the risk of early or delayed cardiotoxicity with the undeniable fact that anti-cancer drugs save lives. Putting this balance centre stage helped to recapitulate all the presentations and discussions, and made the distinction between cardio-oncology and onco-cardiology more than a semantic exercise. Patients should not be denied optimal treatment because of concerns for cardiotoxicity. Excellence in risk: benefit assessment and management will be crucial to the future of cardio-oncology.
